# Efficacy of Sodium-Glucose Cotransporter 2 Inhibitors in Patients With Type 2 Diabetes Mellitus and Nonalcoholic Fatty Liver Disease: A Systematic Review of Randomized Controlled Trials

**DOI:** 10.7759/cureus.86232

**Published:** 2025-06-17

**Authors:** Anas E Ahmed, Nawaf S Alhufayyan, Hadeel F Qadri, Anas F Atiah, Wedad M Alhazmi, Nadim T Alhazemi, Hussam A Zalah, Fahad M Nasser, Rana M Monajid, Massarah G Aljuhani

**Affiliations:** 1 Community Medicine, Jazan University, Jazan, SAU; 2 Internal Medicine, King Fahad Central Hospital, Jazan, SAU; 3 Family Medicine, Jazan Health Cluster, Jazan, SAU; 4 College of Pharmacy, Jazan University, Jazan, SAU; 5 College of Medicine, Jazan University, Jazan, SAU; 6 College of Medicine, King Abdulaziz University, Jazan, SAU; 7 Medicine and Surgery, Almaarefa University, Riyadh, SAU; 8 College of Medicine, Tanta University, Tanta, EGY

**Keywords:** glycemic control, hepatic steatosis, insulin resistance, liver enzymes, liver fat, nafld, nonalcoholic steatohepatitis, randomized controlled trials, sglt2 inhibitors, type 2 diabetes mellitus

## Abstract

Nonalcoholic fatty liver disease (NAFLD) is a common comorbidity in individuals with type 2 diabetes mellitus (T2DM), contributing to increased liver-related complications and cardiovascular risk. Sodium-glucose cotransporter 2 (SGLT2) inhibitors have emerged as potential treatments offering benefits for both metabolic and liver-related outcomes. This systematic review evaluated the efficacy of SGLT2 inhibitors in improving hepatic steatosis, liver enzymes, glycemic control, and liver histology in patients with T2DM and NAFLD. The review was conducted using a structured methodology, including systematic database searches and risk of bias assessments. Included randomized controlled trials (RCTs) investigated various SGLT2 inhibitors, such as dapagliflozin, empagliflozin, ertugliflozin, and ipragliflozin. Most studies showed significant reductions in liver fat content, improvements in serum liver enzymes (alanine aminotransferase (ALT), aspartate aminotransferase (AST), gamma-glutamyl transferase (GGT)), and favorable effects on blood pressure, triglycerides, and body composition. Glycemic control markers, including glycated hemoglobin (HbA1c), fasting glucose, and insulin resistance indices, also improved. One biopsy-based study demonstrated histological improvements, including reduced fibrosis and resolution of nonalcoholic steatohepatitis (NASH). No serious adverse events were reported. These findings suggest SGLT2 inhibitors may offer dual benefits for managing both diabetes and fatty liver disease. Further long-term studies focusing on liver histology as a primary endpoint are needed to confirm these effects.

## Introduction and background

Nonalcoholic fatty liver disease (NAFLD) is the most prevalent chronic liver disorder worldwide, affecting approximately 25% of the global population [1]. Its prevalence is significantly higher in individuals with type 2 diabetes mellitus (T2DM), where it is estimated to affect over 70% of patients [1]. The coexistence of NAFLD and T2DM is clinically significant, as it not only worsens liver-related outcomes but also increases the risk of cardiovascular disease, which remains the leading cause of mortality in this population [2].

The pathophysiological relationship between T2DM and NAFLD is complex and primarily driven by insulin resistance, a key feature of metabolic syndrome. Insulin resistance promotes hepatic lipid accumulation (steatosis), oxidative stress, and inflammation, thereby contributing to the development and progression of NAFLD [3]. Conversely, NAFLD exacerbates systemic insulin resistance, creating a vicious cycle that accelerates the deterioration of both metabolic and hepatic health [3]. Patients with both T2DM and NAFLD are at an increased risk of progressing to nonalcoholic steatohepatitis (NASH), a more aggressive form of the disease characterized by hepatocellular injury, inflammation, and varying degrees of fibrosis [4]. Without intervention, this progression can lead to cirrhosis, liver failure, or hepatocellular carcinoma [4].

Despite the growing burden of NAFLD, there are currently no approved pharmacological treatments specifically targeting this condition. Lifestyle modifications, such as diet, weight loss, and increased physical activity, remain the first-line therapeutic approach. However, achieving and maintaining lifestyle changes is challenging for many patients, and responses to these interventions can be inconsistent [5]. This underscores the urgent need to develop and validate pharmacologic therapies that can effectively improve both hepatic and metabolic outcomes in patients with NAFLD, particularly those with T2DM [6].

Sodium-glucose cotransporter 2 (SGLT2) inhibitors, a class of antidiabetic agents designed to improve glycemic control by promoting urinary glucose excretion, have gained attention for their favorable effects beyond glucose lowering. Clinical trials have demonstrated that SGLT2 inhibitors contribute to weight reduction, blood pressure control, and cardiovascular and renal protection in patients with T2DM [7]. In recent years, emerging evidence suggests that SGLT2 inhibitors may also exert hepatoprotective effects by reducing hepatic fat content, improving serum liver enzyme levels, and attenuating hepatic inflammation and fibrosis [8].

Given these potential advantages, this systematic review aimed to evaluate the efficacy of SGLT2 inhibitors in improving hepatic outcomes among patients with T2DM and NAFLD. By synthesizing data from recent randomized controlled trials (RCTs), this review sought to inform clinical practice and guide future research in this evolving area.

## Review

Methodology

Literature Search Strategy

**Figure 1:**
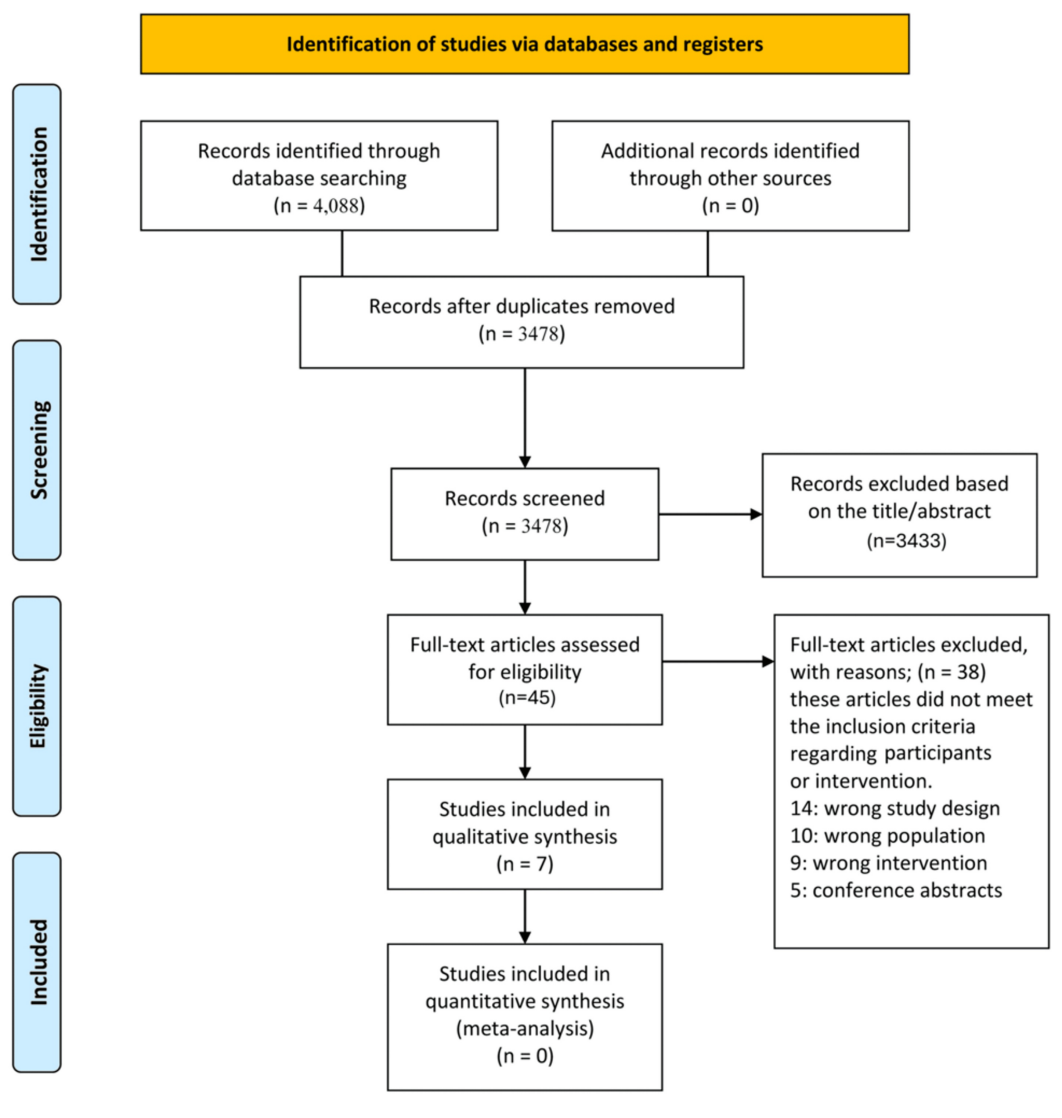
PRISMA flow diagram illustrating the study selection process PRISMA = Preferred Reporting Items for Systematic Reviews and Meta-Analyses.

This systematic review followed the Preferred Reporting Items for Systematic Reviews and Meta-Analyses (PRISMA) guidelines [9]. We searched five databases, namely PubMed, Cochrane Central Register of Controlled Trials (CENTRAL), Virtual Health Library (VHL), Scopus, and Web of Science, up to May 25, 2025. The search combined Medical Subject Headings (MeSH) and free-text terms: ("sodium-glucose transporter 2 inhibitors" OR "SGLT2 inhibitors" OR "dapagliflozin" OR "empagliflozin" OR "canagliflozin" OR "ertugliflozin" OR "ipragliflozin" OR "luseogliflozin" OR "tofogliflozin") AND ("non-alcoholic fatty liver disease" OR "NAFLD" OR "nonalcoholic fatty liver" OR "fatty liver" OR "hepatic steatosis") AND ("diabetes mellitus, type 2" OR "type 2 diabetes" OR "T2DM"). No language or publication date restrictions were applied (Figure 1).

Eligibility Criteria

RCTs published from 2020 onwards were included if they assessed SGLT2 inhibitors in adults with confirmed T2DM and NAFLD. NAFLD diagnosis had to be confirmed by imaging (e.g., ultrasound, computed tomography (CT), magnetic resonance imaging - proton density fat fraction (MRI-PDFF)) or liver biopsy. Interventions involved any SGLT2 inhibitor alone or with standard antidiabetic therapy, compared against placebo, usual care, or non-SGLT2 antidiabetic agents. Studies had to report at least one relevant outcome: liver fat content, liver enzymes (alanine aminotransferase (ALT), aspartate aminotransferase (AST), gamma-glutamyl transferase (GGT)), fibrosis scores, glycemic markers (glycated hemoglobin (HbA1c), fasting plasma glucose), or anthropometric data (body mass index (BMI), weight). Only peer-reviewed full-text articles in English were included. Exclusions were non-RCT designs, animal studies, lack of relevant outcomes, or confounding co-interventions (e.g., insulin, glucagon-like peptide-1 (GLP-1) receptor agonists). Two reviewers independently screened titles and abstracts, then assessed full texts for eligibility. Disagreements were resolved by consensus or by consulting a third reviewer.

Data Extraction

Data were extracted independently using a standardized form, including author, year, country, design, sample size, participant demographics (age, gender), SGLT2 inhibitor type, comparator, treatment duration, and outcomes (liver fat by imaging/histology, liver enzymes, glycemic control, body weight/BMI, fibrosis markers like fibrosis-4 (FIB-4), and inflammatory markers such as tumor necrosis factor alpha (TNF-α), interleukin 6 (IL-6)). Discrepancies were resolved by discussion or third-party adjudication.

Quality Assessment

Two reviewers independently assessed study quality using the Modified Downs and Black Checklist, which evaluates reporting, external validity, bias, confounding, and power. Studies were rated as excellent (26-28), good (20-25), fair (15-19), or poor (≤14). Disagreements were resolved through discussion.

Results

Study Selection

The initial database search yielded 4,088 records from PubMed (n = 322), Cochrane Library (n = 201), VHL (n = 308), Scopus (n = 1,367), and Web of Science (n = 1,890), using the comprehensive search strategy: ("sodium-glucose transporter 2 inhibitors" OR "SGLT2 inhibitors" OR "dapagliflozin" OR "empagliflozin" OR "canagliflozin" OR "ertugliflozin" OR "ipragliflozin" OR "luseogliflozin" OR "tofogliflozin") AND ("non-alcoholic fatty liver disease" OR "NAFLD" OR "nonalcoholic fatty liver" OR "fatty liver" OR "hepatic steatosis") AND ("diabetes mellitus, type 2" OR "type 2 diabetes" OR "T2DM"). After removing duplicates, 3,478 unique records were screened by title and abstract, excluding 3,440 irrelevant articles. Thirty-eight full-text articles were assessed, with 31 excluded due to inappropriate study design (14), population (10), interventions (9), or being conference abstracts (5). Seven studies [10-16] were included for qualitative synthesis; none qualified for meta-analysis.

Characteristics of Included Studies

The seven studies, conducted across China, Iran, Japan, and Thailand, primarily involved patients with T2DM and NAFLD. Shi et al. [14] randomized 78 patients in China to dapagliflozin or other antidiabetics plus metformin over 24 weeks, measuring liver fat content (LFC), pancreatic fat content (PFC), liver enzymes, inflammatory markers, and fibrosis via MRI and blood biomarkers. Shojaei et al. [10] conducted a six-month trial in Iran comparing empagliflozin with standard care in 119 participants, assessing liver steatosis, liver enzymes, and systolic blood pressure. Yoneda et al. [16] performed a double-blind trial in Iran with 70 patients, comparing empagliflozin to placebo over 24 weeks, evaluating liver fat and stiffness via transient elastography, liver enzymes, HbA1c, and BMI (Table 1).

**Table 1:** Summary of included studies evaluating SGLT2 inhibitors in liver disease ALT = alanine aminotransferase; AST = aspartate aminotransferase; ALP = alkaline phosphatase; BMI = body mass index; CAP = controlled attenuation parameter; FIB-4 = fibrosis-4 index; GGT = gamma-glutamyl transferase; HbA1c = glycated hemoglobin; HOMA-IR = homeostatic model assessment for insulin resistance; LAI = liver attenuation index; LSM = liver stiffness measurement; NR = not reported; SBP = systolic blood pressure; TNF-α = tumor necrosis factor alpha; IL-6 = interleukin 6; SGLT2 = sodium-glucose cotransporter 2; US = ultrasound. The arrows indicate direction of change: ↓ decrease, ↑ increase.

Author (Year)	Country	SGLT2 Inhibitor	Duration	Sample Size	Main Outcomes
Shojaei et al. (2025) [10]	Iran	Empagliflozin	24 weeks	119	↓ Liver steatosis (US), ↓ ALT/AST/ALP, ↓ SBP, improved liver grade
Khaliq et al. (2024) [11]	Iran	Ertugliflozin	24 weeks	NR	↓ FIB-4, ↓ ALT/AST/GGT, ↓ HbA1c, ↓ HOMA-IR, ↓ weight/BMI
Phrueksotsai et al. (2021) [12]	Thailand	Dapagliflozin	12 weeks	38	↑ LAI, ↓ ALT, ↓ HbA1c, ↓ BMI, ↓ body fat, anti-inflammatory effect
Takahashi et al. (2022) [13]	Japan	Ipragliflozin	72 weeks	51	↓ Liver enzymes, ↓ fibrosis (biopsy), ↓ type IV collagen 7S, ↓ BMI
Shi et al. (2023) [14]	China	Dapagliflozin	24 weeks	78	↓ Liver/pancreatic fat, ↓ ALT/AST/TNF-α/IL-6, ↓ fibrosis index, weight loss
Takeshita et al. (2022) [15]	Japan	Tofogliflozin	48 weeks	50	↓ Liver fat (MRI), ↓ ALT/AST/GGT, ↓ FIB-4, histological improvement
Yoneda et al. (2021) [16]	Japan	Empagliflozin	24 weeks	70	↓ Liver fat/stiffness (CAP, LSM), ↓ ALT/AST/GGT, ↓ BMI

Japanese studies included Takeshita et al. [15] (50 patients, open-label, 20 weeks, empagliflozin) assessing MRI-PDFF liver fat and metabolic markers and Takahashi et al. [13] (51 patients, multicenter open-label, 72 weeks, ipragliflozin) focusing on biopsy-confirmed histology, including fibrosis and NASH resolution. Khaliq et al. [11] in Iran conducted a 24-week randomized, double-blind, placebo-controlled trial of ertugliflozin, measuring ultrasound liver fat grading, enzymes, lipids, weight, and safety. Phrueksotsai et al. [12] performed a 12-week double-blind placebo-controlled RCT in Thailand with 38 participants, evaluating dapagliflozin effects on liver attenuation index (LAI), body composition, glycemic control, insulin resistance, and adipokines.

Quality Assessment of Included Studies

All studies showed high methodological quality as per the Modified Downs and Black Checklist. Reporting scores ranged from 8 to 10, with Shojaei et al. [10], Takahashi et al. [13], and Phrueksotsai et al. [12] scoring 10 and Khaliq et al. [11] scoring 8. External validity was moderate (score 2/3) across studies. Internal validity scores for bias control were mostly high (score 6/7) except for Takeshita et al. [15] (score 5) and Khaliq et al. [11] (score 4). Confounding control was strongest in Takahashi et al. [13] (score 6), while Khaliq et al. [11] scored lowest with a score of 3. Statistical power was sufficient in six studies [10,12-16], but Khaliq et al. [11] scored 0, raising concerns on reliability due to sample size (Table 2).

**Table 2:** Quality assessment of included randomized controlled trials Quality appraisal of included studies was performed using the Modified Downs and Black Checklist across five domains: reporting (maximum score 10), external validity (maximum score 3), internal validity - bias (maximum score 7), internal validity - confounding (maximum score 6), and power (maximum score 1). Higher scores indicate stronger methodological rigor.

Study	Reporting (0-10)	External Validity (0-3)	Internal Validity - Bias (0-7)	Internal Validity - Confounding (0-6)	Power (0-1)
Shojaei et al. [10]	10	2	6	5	1
Khaliq et al. [11]	8	2	4	3	0
Phrueksotsai et al. [12]	10	2	6	5	1
Takahashi et al. [13]	10	2	6	6	1
Shi et al. [14]	9	2	6	5	1
Takeshita et al. [15]	9	2	5	5	1
Yoneda et al. [16]	9	2	6	5	1

Effects of SGLT2 Inhibitors on Liver Fat, Enzymes, Fibrosis, Glycemic Control, Weight, and Safety

SGLT2 inhibitors consistently reduced liver fat content in NAFLD patients with T2DM. Shi et al. [14] reported significant decreases in both liver and pancreatic fat after 24 weeks of dapagliflozin treatment, accompanied by weight loss in nearly half of the patients. Similarly, Phrueksotsai et al. [12] demonstrated a significant increase in liver attenuation index in the dapagliflozin group, indicating reduced liver fat. Shojaei et al. [10] observed that 17.5% of empagliflozin-treated patients improved from grade 3 to grade 1 steatosis, compared to only 6% in controls. Yoneda et al. [16] and Takeshita et al. [15] also reported reductions in hepatic steatosis, though the latter found no significant change, possibly due to baseline fibrosis variability.

Improvements in liver enzymes, including ALT, AST, and GGT, were noted across most studies, suggesting reduced hepatic inflammation. Shi et al. [14] documented significant ALT reduction along with decreased inflammatory cytokines such as TNF-α and IL-6. Shojaei et al. [10] reported significant declines in ALT, AST, and alkaline phosphatase (ALKP) levels. Both Takeshita et al. [15] and Takahashi et al. [13] observed sustained reductions in liver enzymes and fibrosis indices like FIB-4. Khaliq et al. [11] found significant improvements in enzymes, insulin resistance markers, and lipid profiles. Phrueksotsai et al. [12] demonstrated significant ALT decreases with dapagliflozin versus placebo.

Evidence of antifibrotic effects was present in several studies. Takeshita et al. [15] reported 60% fibrosis improvement based on liver biopsies after tofogliflozin treatment, accompanied by reduced NAFLD activity scores. Takahashi et al. [13] found that 57.1% of patients receiving ipragliflozin achieved at least a one-stage fibrosis reduction compared to 16% in controls, alongside decreased serum fibrosis markers. Khaliq et al. [11] showed significant declines in FIB-4 after ertugliflozin. While Shi et al. [14] suggested fibrosis improvement correlated with decreased inflammatory cytokines, Phrueksotsai et al. [12] and Yoneda et al. [16] did not provide fibrosis-specific data.

All studies consistently reported improved glycemic control with SGLT2 inhibitors. Reductions in fasting plasma glucose and HbA1c were significant, as shown in Shi et al. [14], Shojaei et al. [10], and Yoneda et al. [16]. Takeshita et al. [15] linked glycemic improvements to histological liver benefits, and Khaliq et al. [11] reported enhanced insulin sensitivity alongside glycemic reductions. Phrueksotsai et al. [12] confirmed significant HbA1c declines versus placebo.

Weight and BMI decreased significantly across trials, contributing to improved liver outcomes. Shi et al. [14] observed nearly half of the patients achieving ≥7% weight loss, averaging 6.6%. Takeshita et al. [15] noted a 4.2 kg weight loss at 48 weeks correlated with reductions in steatosis and fibrosis. Takahashi et al. [13], Khaliq et al. [11], and Phrueksotsai et al. [12] also reported meaningful weight and BMI reductions.

SGLT2 inhibitors demonstrated favorable safety profiles, with only mild-to-moderate genital and urinary symptoms reported in some trials (e.g., Takeshita et al. [15]) and no treatment discontinuations due to adverse events. Takahashi [13] observed fewer adverse events with ipragliflozin compared to controls. Shojaei et al. [10], Khaliq et al. [11], and Phrueksotsai et al. [12] reported high completion rates with no severe adverse events, confirming the treatments’ tolerability.

Discussion

This systematic review highlights that SGLT2 inhibitors consistently improve liver-related outcomes in patients with T2DM and NAFLD. Across multiple RCTs, these drugs demonstrated significant reductions in liver fat content, improvements in liver enzyme levels, better glycemic control, and reductions in body weight and BMI [7,8,10-14]. Additionally, some evidence points to beneficial effects on liver fibrosis and inflammation, suggesting that SGLT2 inhibitors may address both metabolic and histological aspects of fatty liver disease [13,15].

One of the most notable findings across the included trials is the significant reduction in hepatic fat content, measured via various imaging modalities such as MRI-proton density fat fraction, controlled attenuation parameter, and liver attenuation index. This reduction is of considerable clinical relevance, as hepatic steatosis is the hallmark of NAFLD and a prerequisite for the development of more advanced liver disease, including NASH, fibrosis, and cirrhosis [1-3]. The observed improvements were most consistently reported with dapagliflozin and ipragliflozin [12-14], although other agents such as empagliflozin and ertugliflozin also demonstrated beneficial effects [10,11].

Improvement in liver enzyme levels (ALT, AST, GGT) was another consistent finding [8,10,12-14]. These enzymes serve as indirect markers of hepatocellular injury and inflammation. Their normalization following SGLT2 inhibitor treatment suggests a reduction in hepatic stress and inflammation, which may, in turn, help halt or reverse disease progression. Additionally, some studies noted reductions in inflammatory markers such as TNF-α and IL-6, supporting a potential anti-inflammatory mechanism of action [13].

Histological evidence, although limited to a few studies, is particularly compelling. In trials that included liver biopsies, improvements in fibrosis stage and resolution of NASH were reported [13,15], supporting the hypothesis that SGLT2 inhibitors may exert disease-modifying effects. These findings mark a significant step forward, as histological improvement is considered the gold standard for assessing NAFLD treatment efficacy [4].

Metabolic benefits of SGLT2 inhibitors, already well-established in the context of T2DM, were also evident. Improvements in glycemic control (HbA1c, fasting plasma glucose, and insulin resistance), body weight, BMI, and visceral adiposity were consistently reported [7,8,10-14]. Since obesity and insulin resistance are key drivers of NAFLD pathogenesis [3,4], these improvements likely contribute to the hepatic benefits observed.

Notably, the safety profile of SGLT2 inhibitors in these studies was favorable. No serious adverse events were attributed to the interventions, and most side effects were mild and manageable, such as genitourinary infections [7,8,10-12]. This tolerability further supports the utility of SGLT2 inhibitors in clinical practice [5,6].

Despite these encouraging findings, several limitations must be acknowledged. First, the overall number of high-quality RCTs remains limited, with relatively small sample sizes and short durations of follow-up. Longitudinal studies assessing long-term hepatic outcomes, including progression to cirrhosis or liver-related mortality, are needed. Second, most included trials were conducted in Asian populations [10-14], potentially limiting the generalizability of findings to other ethnic groups with different NAFLD phenotypes and risk profiles. Third, although imaging and biomarker-based endpoints were widely used, only a few studies incorporated liver biopsies [13,15], which are essential for definitive conclusions regarding histological improvement [9].

## Conclusions

This review highlighted the promising role of SGLT2 inhibitors as a therapeutic option for patients with T2DM and coexisting NAFLD. The consistent improvements observed in liver fat content, liver enzymes, metabolic parameters, and in some cases, histological markers suggest that these agents offer more than just glycemic control; they may also help address the underlying hepatic pathology associated with fatty liver disease. Importantly, these benefits were achieved alongside favorable safety profiles, making SGLT2 inhibitors a potentially valuable addition to the treatment strategy for this patient group. However, due to variations in study design and limited long-term data, further research is needed to confirm these findings, particularly regarding their impact on liver fibrosis and disease progression. Overall, SGLT2 inhibitors represent a promising avenue for integrated metabolic and hepatic management in clinical practice.
